# Expression of miR159 Is Altered in Tomato Plants Undergoing Drought Stress

**DOI:** 10.3390/plants8070201

**Published:** 2019-07-02

**Authors:** María José López-Galiano, Inmaculada García-Robles, Ana I. González-Hernández, Gemma Camañes, Begonya Vicedo, M. Dolores Real, Carolina Rausell

**Affiliations:** 1 Department of Genetics, University of Valencia, Burjassot, 46100 Valencia, Spain; 2 Plant Physiology Area, Biochemistry and Biotechnology Group, Department CAMN, University Jaume I, 12071 Castellón, Spain

**Keywords:** *Solanum lycopersicum*, drought, Colorado potato beetle, miR159, MYB transcription factors, *P5CS*, proline, putrescine

## Abstract

In a scenario of global climate change, water scarcity is a major threat for agriculture, severely limiting crop yields. Therefore, alternatives are urgently needed for improving plant adaptation to drought stress. Among them, gene expression reprogramming by microRNAs (miRNAs) might offer a biotechnologically sound strategy. Drought-responsive miRNAs have been reported in many plant species, and some of them are known to participate in complex regulatory networks via their regulation of transcription factors involved in water stress signaling. We explored the role of miR159 in the response of *Solanum lycopersicum* Mill. plants to drought stress by analyzing the expression of sly-miR159 and its target SlMYB transcription factor genes in tomato plants of cv. Ailsa Craig grown in deprived water conditions or in response to mechanical damage caused by the Colorado potato beetle, a devastating insect pest of Solanaceae plants. Results showed that sly-miR159 regulatory function in the tomato plants response to distinct stresses might be mediated by differential stress-specific MYB transcription factor targeting. sly-miR159 targeting of SlMYB33 transcription factor transcript correlated with accumulation of the osmoprotective compounds proline and putrescine, which promote drought tolerance. This highlights the potential role of sly-miR159 in tomato plants’ adaptation to water deficit conditions.

## 1. Introduction

Climate change due to increasing concentration of CO_2_ in the atmosphere is leading to rising temperatures, altered rainfall patterns, and more frequent and severe drought episodes [1], which negatively impact crop production. Therefore, gaining knowledge about how plants regulate their adaptation to stress is critical to find ways to enhance plant performance in eventually drier environments. 

To cope with drought, plants activate a complex cascade of events at the cellular level that include extensive metabolic and gene transcriptional reprogramming to protect cells from osmotic stress, and limit water loss. The response of plants to drought stress involves genes related to diverse functional categories such as genes encoding proteins participating in the direct protection of essential proteins and membranes (osmoprotectants, free radical scavengers, etc.), genes encoding membrane transporters and ion channels that promote water uptake, and genes encoding stress related regulatory proteins such as kinases and transcription factors belonging to the V-myb myeloblastosis viral oncogene homolog (MYB), basic-helix-loop-helix (bHLH), basic region/leucine zipper (bZIP), NAM, ATAF1/2, and CUC (NAC), and APETALA2/ethylene-responsive element binding protein (AP2/EREBP) families [2].

The phytohormone Abscisic acid (ABA) coordinates the plant’s response to reduced water availability by modulating the expression of some of the drought responsive genes [3]. Interestingly, microRNAs (miRNAs) have been recently reported to mediate drought tolerance by post-transcriptionally regulating drought-responsive genes, some of which are known to be controlled by ABA signaling pathways [4]. An example of such intricate regulatory network is provided by miR159, which in *Arabidopsis* germinating seeds, has been reported to be induced by ABA and drought treatments, and promote transcript cleavage of the ABA positive regulators MYB33 and MYB101 transcription factors, thereby playing a key role in ABA response [5]. 

The miR159 family is highly conserved among monocot and dicot plants, but in plants undergoing drought, miR159’s relative abundance varies in a tissue- and species-specific manner. For instance, miR159 was reported to be up-regulated by drought stress in *Arabidopsis* [6], and maize [7], but down-regulated in cotton [8], and potato [9], whereas in barley and alfalfa, miR159 was down-regulated in roots and up-regulated in leaves in response to drought stress [10,11]. Pegler et al. [12] proposed that the differential miRNA abundance across species following drought or salt stress exposure might be in part due to differential distribution of regulatory transcription factor binding sites within the putative promoter region of the miRNA gene, which encodes the highly conserved, stress-responsive miRNA.

To expand our knowledge on the miR159 regulatory network involved in tomato plants’ response to drought stress, in the present work we analyzed the expression of miR159 and its predicted target genes in tomato plants of *Solanum lycopersicum* Mill. cv. Ailsa Craig undergoing drought stress, in which we previously reported that ABA hormone is accumulated after water deprivation [13].

## 2. Results and Discussion

### 2.1. Expression of miR159 in Tomato Plants Undergoing Drought Stress

**Figure 1:**
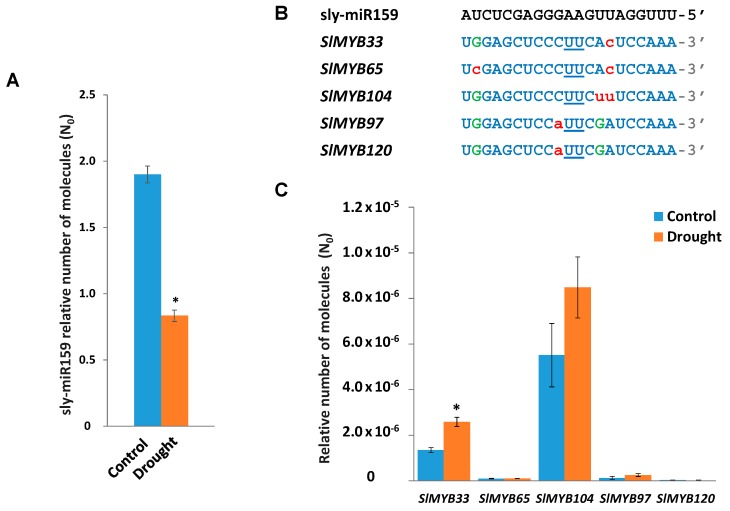
RT-qPCR analysis of sly-miR159 expression and its *MYB* predicted targets in tomato plants undergoing drought stress. (**A**) RT-qPCR analysis of sly-miR159 expression in control tomato plants and tomato plants following 7-day water deprivation. (**B**) Nucleotide sequence of sly-miR159-binding sites in tomato GAMYB-like transcripts. Nucleotides in the cleavage site are underlined, lower-case red letters indicate mismatches to sly-miR159, and G:U pairing is shown in uppercase green letters. (**C**) RT-qPCR analysis of *SlMYB33*, *SlMYB65*, *SlMYB104*, *SlMYB97,* and *SlMYB120* genes expression in control tomato plants and tomato plants following 7-day water deprivation. In panels (A) and (C), data shown are the mean of three independent experiments ± standard error (SE). Asterisk indicates that differences between means of control and undergoing drought stress tomato plants were statistically significant (Student’s *t*-test, *p* < 0.05).

To assess miR159 expression in tomato plants of *Solanum lycopersicum* Mill. cv. Ailsa Craig following a seven-day water deprivation, we analyzed sly-miR159 (GenBank: 102464332) transcript levels by RT-qPCR in control tomato plants and plants undergoing drought stress. Results showed significantly reduced expression of sly-miR159 in response to stress (Figure 1A). However, in recent high-throughput sequencing studies performed by Liu et al. [14,15], miR159 was not found among the miRNAs differentially expressed after 10 days of drought stress in a sensitive and a tolerant tomato cultivar. This apparent discrepancy with our results might be due to the differential experimental conditions or techniques used to measure miRNA expression, but is most probably due to the fact that the tomato cultivars were different, since it has been reported that miRNAs respond to environmental stresses in a genotype-dependent manner [16]. As in plants, most miRNAs negatively regulate their target genes, we hypothesized that sly-miR159 gene targets that are upregulated in tomato plants grown in water-limited conditions in our experimental conditions may play beneficial roles in the adaptive responses to drought stress. 

In *Arabidopsis*, a clade of seven closely related *GAMYB*-like genes (*MYB33*, *MYB101*, *MYB65*, *MYB81*, *MYB97*, *MYB104*, and *MYB120*) share a conserved putative miR159-binding site [17]. The *GAMYB*-like genes encode a highly conserved family of R2R3-type MYB domain transcription factors that are regulated by Gibberellic acid (GA) and ABA and participate in the GA signaling pathway [18]. Recent studies in potato plants highlight the involvement of miR159 and its targets *GAMYB*-like genes in the response of this species to water stress [9]. Using psRNATarget software [19] we identified the following putative *GAMYB*-like transcription factor genes that are sly-miR159 targets in tomato: *SlMYB33* (Solyc01g009070.2.1), *SlMYB65* (Solyc06g073640.2.1), *SlMYB104* (Solyc11g072060.1.1), *SlMYB97* (Solyc10g019260.1.1), and *SlMYB120* (Solyc01g090530.1.1). Figure 1B shows the nucleotide sequence of the sly-miR159-binding sites in the tomato *SlMYB* transcripts identified, which strongly resemble those found in *AtMYB* transcripts targeted by miR159 in *Arabidopsis* [20].

Li et al. [21] identified 127 *MYB* genes in the tomato genome and classified the corresponding proteins into 18 subgroups based on domain similarity and phylogenetic topology, and suggested that conserved motifs outside the MYB domain might reflect their functional conservation. SlMYB33, SlMYB65, and SlMYB104 proteins cluster in subgroup 12, in which the three of them are the only ones (out of the thirteen subgroup members) sharing the conserved motifs 14 and 15 outside the MYB domain. SlMYB97 and SlMYB120 proteins constitute subgroup 15, which is composed only by these two MYB proteins that have no conserved motifs outside the MYB domain. 

We analyzed the expression of sly-miR159 *MYB* predicted targets in control tomato plants and plants undergoing drought stress by RT-qPCR (Figure 1C). Only *SlMYB33* gene showed statistically significant induction in water-stressed tomato plants, exhibiting an opposite pattern of expression relative to that of sly-miR159, which suggests that this *MYB* gene may be regulated by sly-miR159 in tomato plants in response to drought stress. In line with this hypothesis, in potato plants in which the *CBP80* gene encoding a protein involved in RNA processing was silenced, improved tolerance to water stress was correlated with decreased levels of miR159 and enhanced *MYB33* gene expression [22].

**Figure 2:**
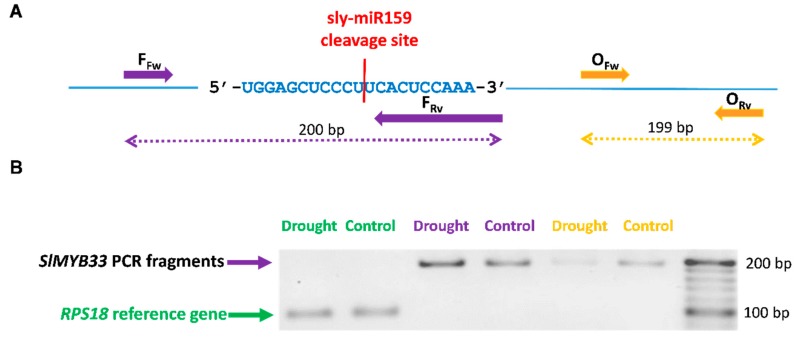
RT-PCR amplification of SlMYB33 mRNA fragments. (**A**) Nucleotide sequence of sly-miR159-binding sites in SlMYB33 transcripts. Bar in red depicts the putative cleavage site and arrows indicate the annealing positions of primer pair OFw and ORv, outside the sly-miR159-binding region, and primer pair FFw and FRv, flanking the putative cleavage site in sly-miR159-binding region. (**B**) RT-PCR analysis of SlMYB33 small RNA fragments in control tomato plants and tomato plants following 7-day water deprivation using primers OFw and ORv, or FFw and FRv. RPS18 gene expression was used as normalization control. For each sample, three biological replicates were pooled and analyzed.

To further assess the involvement of sly-miR159 in the regulation of *SlMYB33* gene expression under drought stress, we aimed at analyzing *SlMYB33* cleavage fragments. We designed two pairs of primers to amplify *SlMYB33* mRNA fragments in small RNA samples isolated from total RNA of control tomato plants and tomato plants following a seven-day water deprivation (Materials and Methods, Section 4.3). Figure 2A shows the annealing positions of both pairs of PCR primers. The primer pair O_Fw_ and O_Rv_ anneals to sequences within a *SlMYB33* mRNA region downstream of the predicted sly-miR159-binding site, yielding a 199 bp *SlMYB33* amplification product. The primer pair F_Fw_ and F_Rv_ anneals to sequences flanking the putative cleavage site in the predicted sly-miR159-binding region, yielding a 200 bp *SlMYB33* amplification product only when the SlMYB33 mRNA is not cleaved at the sly-miR159 cleavage site. Therefore, we hypothesized that if sly-miR159 is not involved in the regulation of *SlMYB33* gene expression of the same amplification patterns of control vs. drought, then small RNA samples with both primer pairs would be expected. Figure 2B shows the results obtained in the RT-PCR amplifications using the two pairs of primers. Lower amounts of amplification products were obtained using primers O_Fw_ and O_Rv_ in tomato plants grown under water scarcity compared to control plants. In contrast, higher amount of PCR amplified product was observed in drought-stressed tomato plants than in control tomato plants using primers F_Fw_ and F_Rv_. Collectively, these results support targeted cleavage of *SlMYB33* transcripts by sly-miR159 that might participate in the transcriptional regulation of the tomato plants’ response to drought stress.

**Figure 3:**
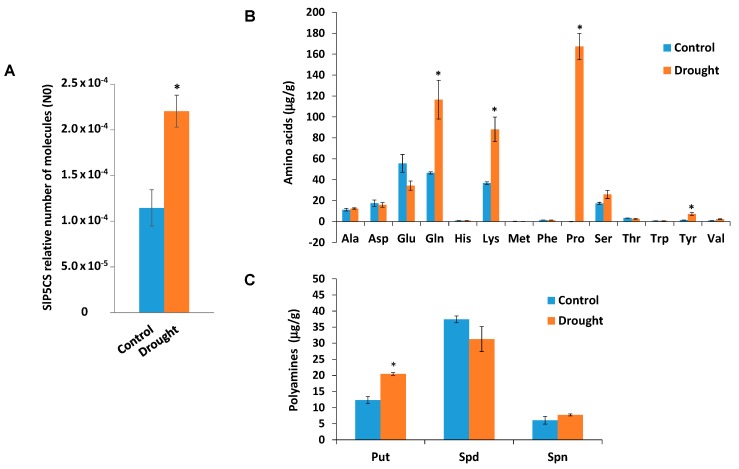
Analysis of *SlP5CS* gene expression, and amino acid and polyamines in tomato plants undergoing drought stress. (**A**) RT-qPCR analysis of *SlP5CS* expression in control tomato plants and tomato plants following 7-day water deprivation. (**B**) Amino acids levels upon drought treatment. Amino acids levels are expressed in µg/g DW. (**C**) Polyamines levels upon drought treatment. Polyamines levels are expressed in µg/g DW. Put (putrescine), Spd (Spermidine), Spn (Spermine). Tomato leaves were collected from plants that were properly irrigated (Control) or deprived of water 1 week (Drought). Data shown are the mean of three independent experiments ± standard error (SE). Asterisk indicates that differences between means of control and undergoing drought stress tomato plants were statistically significant (Student’s *t*-test, *p* < 0.05).

Interestingly, Qin et al. [23] proposed that MYB33 transcription factor may enhance drought tolerance by means of promoting osmotic pressure balance reconstruction and reactive oxidative species (ROS) scavenging, since ectopic over-expression of wheat *MYB33* gene in *Arabidopsis* induced the expression of *AtP5CS* and *AtZAT12* genes involved in proline synthesis and ascorbate peroxidase synthesis, respectively. Accordingly, we observed an induction of *SlP5CS* gene expression and a remarkable increase in proline levels relative to other amino acids in tomato plants grown in water-shortage conditions compared to irrigated control plants (Figure 3A,B), suggesting that sly-miR159 might participate in the tomato plants’ adaptive response to drought stress via induction of *SlMYB33* transcription factor gene expression. Nevertheless, further research is needed to demonstrate whether the sly-miR159-SlMYB33 pathway is necessary for drought tolerance in the tomato cultivar Ailsa Craig.

Tonon et al. [24] proposed a strong metabolic coordination between polyamines and proline pathways in response to osmotic stresses. Therefore, we analyzed polyamine levels in tomato plants undergoing drought stress and non-stressed control plants (Figure 3C), and results showed increased accumulation of putrescine, a polyamine reported to have a role in protecting plants during water-deficient conditions, as well as oxidative stress [25]. In wheat, Pál et al. [26] recently described that ABA pre-treatments induced the expression of *P5CS* gene and enhanced the accumulation of putrescine. Authors suggested that the connection between polyamine metabolism and ABA signaling may control the regulation and maintenance of polyamine and proline levels under osmotic stress conditions in wheat seedlings.

### 2.2. Assessment of sly-miR159 Stress-Specific Targeting of SlMYB33

**Figure 4:**
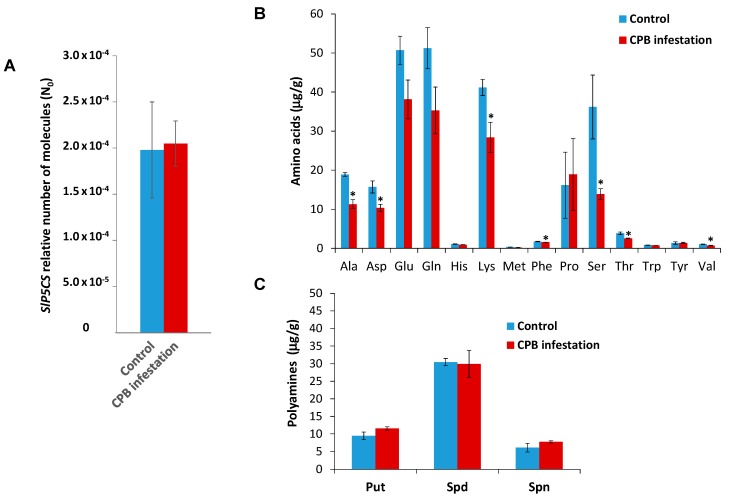
Analysis of *SlP5CS* gene expression, and amino acid and polyamines in tomato plants infested by Colorado potato beetle (CPB) larvae. (**A**) RT-qPCR analysis of *SlP5CS* expression in control tomato plants and tomato plants infested by CPB larvae. (**B**) Amino acids levels upon CPB infestation. Tomato leaves were collected from non-infested plants (Control) or plants infested by CPB. Amino acids levels are expressed in µg/g DW. (**C**) Polyamines levels upon CPB larvae infestation. Tomato leaves were collected from non-infested plants (Control) or plants infested by CPB. Polyamines levels are expressed in µg/g DW. Put (putrescine), Spd (Spermidine), Spn (Spermine). Data shown are the mean of three independent experiments ± standard error (SE). Asterisk indicates that differences between means of control and undergoing drought stress tomato plants were statistically significant (Student´s *t*-test, *p* < 0.05).

To ascertain whether *SlMYB33* targeting by sly-miR159 is stress-specific, we analyzed the expression of sly-miR159 and its predicted MYB target genes in tomato plants attacked by the coleopteran insect pest Colorado potato beetle (CPB), in which we previously reported that, as opposed to tomato plants undergoing drought stress, ABA was not accumulated [13]. In the present work, neither *SlP5CS* gene expression were induced, nor were increased proline and putrescine levels observed in infested tomato plants compared to tomato control plants (Figure 4), corroborating that the plants’ response to this biotic stress is different from the plant response to water stress. 

**Figure 5:**
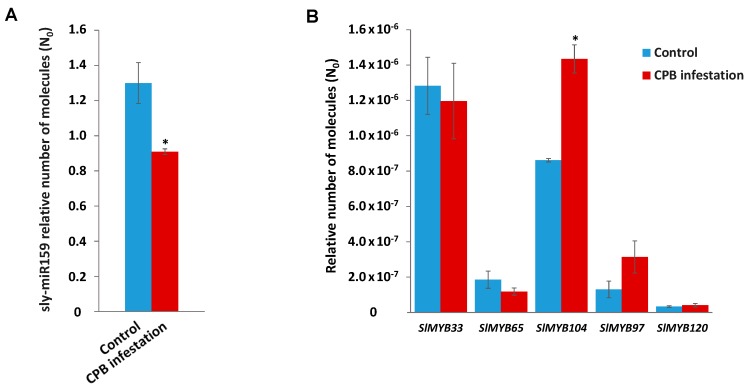
RT-qPCR analysis of sly-miR159 expression and its *MYB* predicted targets in tomato plants infested by CPB larvae. (**A**) RT-qPCR analysis of sly-miR159 expression in control tomato plants and tomato plants infested by CPB larvae. (**B**) RT-qPCR analysis of *SlMYB33*, *SlMYB65*, *SlMYB104*, *SlMYB97,* and *SlMYB120* genes expression in control tomato plants and tomato plants upon CPB larvae infestation. In panels (A) and (B), data shown are the mean of three independent experiments ± standard error (SE). Asterisk indicates that differences between means of control tomato plants and tomato plants infested by CPB larvae were statistically significant (Student´s *t*-test, *p* < 0.05).

Intriguingly, as it was observed in plants deprived of water, in infested tomato plants, sly-miR159 was significantly down-regulated compared to non-infested control plants (Figure 5A). However, in plants attacked by CPB, among sly-miR159 putative *MYB* targets, only the *SlMYB104* transcript factor gene was significantly up-regulated (Figure 5B), suggesting that sly-miR159 might be regulating this specific MYB transcription factor in response to CPB damage.

In contrast, correlating with the lack of proline and putrescine accumulation, no variation was detected in *SlMYB33* transcription factor gene expression. This suggests that the specificity of the stress response regulated by sly-miR159 might, at least in part, rely on the distinct *MYB* transcription factor transcript that the sly-miR159 sRNA specifically regulates under each stress condition. It has been proposed that additional factors other than complementarity and cleavage, such as target accessibility and secondary structure, RNA binding proteins, and target site context may modulate silencing efficiency [27], which might lie at the root of the stress specific miR159 regulation of MYB transcription factors, and deserve further research.

## 3. Conclusions

Overall, the results obtained in this work show the potential involvement of sly-miR159 in the tomato plants’ response to different stresses through stress-specific *MYB* transcription factor targeting. Under drought-stress, sly-miR159 targeting of *SlMYB33* correlates with induction of *SlP5CS* gene expression and accumulation of the osmoprotective compounds proline and putrescine, pointing to the possible participation of this miR in the regulation of drought stress tolerance. Understanding the regulatory network underlying drought stress response may provide new biotechnological approaches to generate plants better adapted to dry environments. Our results support that in addition to using *SlMYB33* transcription factor as a biotechnological target for metabolic engineering by ectopic expression, *SlMYB33* gene expression reprogramming by sly-miR159 might develop into a useful system to improve plant drought tolerance in tomato plants.

## 4. Materials and Methods

### 4.1. Plants

Thirty-day-old tomato plants of *Solanum lycopersicum* Mill. cv. Ailsa Craig (four-week-old) were grown from germinated seeds in a growth chamber under the following environmental conditions: 16/8 h light/night cycle, 26/18 °C day/night temperature cycle, and 60% relative humidity (RH). Seeds were irrigated twice a week with distilled water during the first week, and with Hoagland solution thereafter [28].

For drought stress experiments, thirty-day-old tomato plants were deprived of water for 7 days, and leaf tissue from 3rd and 4th leaves was collected, frozen in liquid nitrogen, and stored at −80 °C. Leaf tissue from 3rd and 4th leaves of irrigated plants was also collected as control.

For Colorado potato beetle (CPB) infestation, 15 CPB larvae of different developmental stages were placed on the 3rd and 4th leaves of thirty-day-old tomato plants. When necessary, non-cooperative larvae (molting or not eating) were removed and substituted. Leaf tissue left after 3 h of CPB feeding and that of the non-infested control plants were harvested, frozen in liquid nitrogen, and stored at −80 °C.

### 4.2. Total RNA Isolation and RT-qPCR Analysis

Total RNA was isolated from leaves of control tomato plants and plants undergoing drought stress or CPB infestation using RiboPure Kit (Ambion, Cat. No. AM1924), following the manufacturer’s protocol. TURBO DNA-free kit (Ambion, Cat. No. AM1907) was used to remove contaminating genomic DNA from RNA preparations and RNA quality was evaluated by 1% agarose gel electrophoresis and quantified spectrophotometrically (NanoDrop 2000, Thermo Scientific, Waltham, MA, USA).

RT-qPCR amplification was performed using SYBR Premix Ex Taq II (Takara).

For sly-miR159 amplification 1 µg of RNA was polyadenylated in a final volume of 10 μL, including 1 μL of 10x poly(A) polymerase buffer, 1 mM of ATP, and 1 unit of poly(A) polymerase (New England Biolabs, Ipswich, MA, USA), and incubated at 37 °C for 15 min and then at 65 °C for 20 min. Polyadenylated RNA was reverse transcribed to complementary DNA (cDNA) using the Universal RT-primer (Integrated DNA Technologies, Coralville, IA, USA) described in Balcells et al. [29] (5′-CAGGTCCAGTTTTTTTTTTTTTTTVN-3′, where V is A, C, and G, and N is A, C, G, and T). Reverse transcription reaction was performed using PrimeScript™ RT reagent Kit (Takara) in a final volume of 10 μL, including 2 μL of 5X PrimeScript™ Buffer, 0.5 μL of PrimeScript™ RT Enzyme Mix I, and 1 μM of Universal RT-primer, and it was incubated at 37 °C for 15 min followed by enzyme inactivation at 85 °C for 5 s. Forward and reverse primers for miRNA RT-qPCR amplification were designed according to Balcells et al. [29] (Table 1).

For *SlMYB33*, *SlMYB65*, *SlMYB104*, *SlMYB97*, *SlMYB120,* and *SlP5CS* transcript amplification, the PrimeScript™ RT reagent kit (Takara) was used for cDNA synthesis according to the manufacturer’s protocol using 50 ng/µL oligo(dT) (Promega), and 2.5 μM random hexamers (Applied Biosystems). Ten ng cDNA, and gene specific forward (F) and reverse (R) primers (Table 1), designed with PRIMER3PLUS software [30], were used.

A StepOnePlus Real-Time PCR system (Applied Biosystems) was used, under the conditions recommended by the manufacturer, and the cycling parameters were: Initial polymerase activation step at 95 °C for 30 s, 40 cycles of denaturation at 95 °C for 5 s, annealing, and elongation at 60 °C for 30 s. For each sample, three biological replicates (with 3 technical replicates each) were analyzed. Relative-fold calculations were made using *RPS18* (ribosomal protein S18, GeneBank: 3950409) gene to normalize gene expression, and *U6* snRNA gene (GenBank: X51447.1) to normalize sly-miR159 expression (Table 1). LingReg software [31] was employed for the analysis of RT-qPCR experiments and data were analyzed by Student’s *t*-test for statistically significant differences (*p* < 0.05).

Each biological sample from the 3rd and 4th leaves of plants undergoing drought stress and their corresponding controls consisted of a pool of total RNA from 25 plants. Biological samples in CPB infestation experiments and their corresponding controls also consisted of a pool of total RNA from 25 plants.

### 4.3. Small RNA Isolation and RT-PCR Analysis

The small RNA fraction in total RNA samples of control tomato plants and tomato plants following 7-day water deprivation was isolated using Nucleospin^®^ miRNA (Macherey-Nagel, Bethlehem, PA, USA) following the manufacturer’s instructions.

For *SlMYB33* small mRNA amplification, the PrimeScript™ RT reagent kit (Takara, Shiga, Japan) was used for cDNA synthesis according to the manufacturer’s protocol using 50 ng/µL oligo(dT) (Promega, Madison, WI, USA) and 2.5 μM random hexamers (Applied Biosystems, Waltham, MA, USA), 10 ng cDNA, and gene specific forward (F) and reverse (R) primers (Table 2), designed with PRIMER3PLUS software [30]. *RPS18* (ribosomal protein S18, GeneBank: 3950409) was used as a reference gene.

The cycling parameters were as follows: Initial polymerase activation step at 95 °C for 30 s, 40 cycles of denaturation at 95 °C for 5 s, annealing, and elongation at 60 °C for 30 s. For each sample, three biological replicates were pooled and analyzed. Five microliters of the reaction volume were separated in a 3% agarose gel.

### 4.4. Amino Acids and Polyamines Quantification

Leaves were recollected after stress condition and frozen in liquid N_2_, ground, and lyophilized.

For amino acids analysis, dry tissue (0.1 g) was homogenized with 800 µL of extraction solution: 400 µL of distilled water, 200 µL of chloroform, and 200 µL of methanol per sample. Moreover, a mixture of internal standards was added prior to extraction (100 ng of Phe ^13^C_9_^15^N and 100 ng of Thr ^13^C_4_^15^N). Samples were filtered, and a final concentration of 1 mM perfluoroheptanoic acid as ion-pairing reagent was added to each sample. A 20 μL aliquot was injected into a high-performance liquid chromatography system (HPLC) with an XSelect HSS C18 column (5 μm 2.1 × 100 mm) which was interfaced with a triple quadrupole mass spectrometer (TQD, Waters, Manchester, UK).

Polyamine analysis was conducted according to the method described by Sánchez-López et al. [32], using as internal standards a mixture of [^13^C_4_]-putrescine and 1,7-diamineheptane. To analyze each condition, ten independent biological replicates per sample were generated and three independent experiments were conducted.

## Figures and Tables

**Table 1:** Primers used to analyze by RT-qPCR sly-miR159, *SlMYB,* and *SlP5CS* gene expression in tomato plants.

Gene	Forward Primer (5′-3′)	Reverse Primer (5′-3′)	Product Size (bp)
sly-miR159	CGCAGTTTGGATTGAAGGGAG	CAGGTCCAGTTTTTTTTTTTTTTTTAGAG	50
*SlMYB33*	TATGGGCATCCAGTCTCTCC	TGGGACTGGAAAAGATCGTC	199
*SlMYB65*	TCTGCTGCATCGGTGTTTAG	TCTGGCCTGGGACAGATAAG	164
*SlMYB104*	TTTCGGAATTGTTTGGAAGC	TGAAGAAGTTGCCGACAATG	110
*SlMYB97*	CATGTCCCCTTGGAAGATTTAG	CTAGTGGCAAAGCAAAGTCATC	181
*SlMYB120*	CACATTCCAGTCCAAACCAAC	CCTAGGTCGGAAGCACTGAG	116
*SlP5CS*	TGCTCAACAGGCCGGATATG	AAAGTGTGACCAAGGGGCTC	126
*U6* snRNA	GGGGACATCCGATAAAATTGGAAC	TGGACCATTTCTCGATTTGTGC	88
*RPS18*	GGGCATTCGTATTTCATAGTCAGAG	CGGTTCTTGATTAATGAAAACATCCT	105

**Table 2:** Primers used to analyze *SlMYB33* small transcript fragments by RT-PCR in tomato plants annealing to a region outside the predicted sly-miR159 binding site in *SlMYB33* mRNA (O_Fw_, O_Rv_) or flanking the putative cleavage site within the predicted sly-miR159 binding site in *SlMYB33* mRNA (F_Fw_, F_Rv_).

Primer Pair	Forward Primer (5′-3′)	Reverse Primer (5′-3′)	Product Size (bp)
O_Fw_, O_Rv_	TATGGGCATCCAGTCTCTCC	TGGGACTGGAAAAGATCGTC	199
F_Fw_, F_Rv_	ATGACGGTTCTTTGCTTGCT	CTGTCTGGTTTTGGAGTGAAGG	200
*RPS18*_FW_, RPS18_RV_	GGGCATTCGTATTTCATAGTCAGAG	CGGTTCTTGATTAATGAAAACATCCT	105
